# Targeting the M1 muscarinic acetylcholine receptor in Alzheimer’s disease

**DOI:** 10.1042/NS20210004

**Published:** 2022-04-21

**Authors:** Louis Dwomoh, Gonzalo S. Tejeda, Andrew B. Tobin

**Affiliations:** The Centre for Translational Pharmacology, Institute of Molecular, Cell and Systems Biology, College of Medical, Veterinary and Life Sciences, University of Glasgow, Glasgow, United Kingdom

**Keywords:** acetylcholine, allosteric regulation, Alzheimer's disease, muscarinic receptor, orthosteric

## Abstract

Alzheimer’s disease (AD) remains a major cause of morbidity and mortality worldwide, and despite extensive research, only a few drugs are available for management of the disease. One strategy has been to up-regulate cholinergic neurotransmission to improve cognitive function, but this approach has dose-limiting adverse effects. To avoid these adverse effects, new drugs that target specific receptor subtypes of the cholinergic system are needed, and the M1 subtype of muscarinic acetylcholine receptor (M1-mAChR) has been shown to be a good target for this approach. By using several strategies, M1-mAChR ligands have been developed and trialled in preclinical animal models and in human studies, with varying degrees of success. This article reviews the different approaches to targeting the M1-mAChR in AD and discusses the advantages and limitations of these strategies. The factors to consider in targeting the M1-mAChR in AD are also discussed.

## Introduction

### Alzheimer’s disease

Alzheimer’s disease (AD) is a progressive neurodegenerative disorder which is characterised by accumulation of β-amyloid peptide (Aβ) and neurofibrillary tangles (NFTs) [1]. In addition to these neuropathological features, there is significant disruptions in several neurotransmitter systems, particularly in cholinergic neurotransmission, which leads to cognitive impairments [2]. Disrupted cholinergic neurotransmission in AD is also associated with behavioural disturbances, including agitation, apathy, sleep disorders, and depression [3,4].

AD accounts for more than 60% of all dementia cases, and with increases in life expectancy across the world, AD cases are expected to rise. Estimates show that approximately 50 million people live with AD worldwide, with cases expected to triple by 2050, if there are no research and medical breakthroughs to slow down the progression or cure the disease [5]. Furthermore, the disease presents with significant economic, social and health impacts. For instance, in 2020, 11 million family members and carers of persons living with AD provided over 15 billion hours of unpaid care, estimated at $256 billion [5]. In the U.K., unpaid care makes up approximately 40% of the total cost of dementias, including AD [6].

### Current treatments for AD

As a complex, multifactorial disease, there is currently no cure for AD. The currently approved and clinically available medications for AD can be grouped into three:

#### Medications to manage behavioural disturbances

These include antidepressants and antipsychotics to manage behavioural and psychological symptoms. Risperidone is an antipsychotic approved for short-term use in refractory severe agitation or psychosis [7]. It is recommended as one of the last resorts, considering that it presents with adverse effects, including cognitive decline [8].

#### Medications to improve cognitive function

- Acetylcholinesterase inhibitors (AChEIs), including donepezil, galantamine, and rivastigmine inhibit the breakdown of acetylcholine (ACh) in the synapse, thereby enhancing cholinergic neurotransmission and resulting in short- and long-term reduction in decline in cognitive function in patients with mild to moderate AD [9]. However, AChEIs cause whole-body up-regulation of cholinergic signalling, resulting in dose-limiting adverse effects, including nausea, diarrhoea, salivation, cramping, and reduced heart rate [10].
- NMDA receptor antagonists, including memantine slows the decline in cognitive function in patients with moderate to severe AD [11]. Other trials have shown an enhanced benefit in cognitive function with reduced rate of clinical worsening in AD patients who are on a combination treatment of donepezil and memantine [12]. There is evidence for adverse effects of memantine, including somnolence [13].

#### AD-modifying medications

Until recently, none of the medications for AD had disease-modifying effects. In mid-2021, the U.S. FDA granted an accelerated approval for Aducanumab, a monoclonal antibody that targets the Aβ protein, as the first disease-modifying treatment for AD [14,15]. This approval was significant in many ways, including the successful targeting of one of the most prominent pathological markers of AD to improve cognition. The use of Aducanumab has several limitations, including cost, reported cases of asymptomatic brain swellings, and being only approved for use in persons with mild cognitive impairment (MCI) or mild dementia due to AD [16]. There is also the need to verify the clinical benefits of the drug in future confirmatory trials [15]. Other disease-modifying AD drugs are in various stages of development, including Elli Lilly’s Donanemab, another antibody that targets the modified form of deposited Aβ [17].

Taken together, most of the existing medications for AD primarily treat the symptomatic presentations, with dose-limiting adverse effects. It is therefore important to explore and validate novel therapeutic targets to slow down or reverse cognitive decline and/or modify progression of AD without significant adverse effects. One of such strategies is to target the disrupted cholinergic neurotransmission with drugs that are selective for their target.

## The cholinergic system in AD

### The cholinergic system

The cholinergic system consists of the nicotinic (nAChR) and muscarinic (mAChR) receptors, together with the neurotransmitter ACh. In the mammalian nervous system, several groups of cholinergic-expressing neurons exist, including those within the spinal cord, brainstem, basal forebrain, and the periphery [18]. The cholinergic neurons of the basal forebrain are important components of cholinergic signalling in the CNS, being the sources of the major cholinergic innervations of the hippocampus, cortex, and amygdala [18]. Thus, they play important roles in cognitive function, as well as in the regulation of memory and attention [18], and degeneration of these cholinergic neurons has been shown to precede and predict cognitive impairment in neurodegenerative disorders, including AD [19].

The neurotransmitter, ACh is primarily synthesised in the nerve terminal cytoplasm from acetyl coenzyme A and choline, in a reaction catalysed by choline acetyltransferase (ChAT). It is then released into the synapse and binds to the two classes of receptors; the G-protein coupled muscarinic receptors and the ionotropic nicotinic receptors, to transmit signals from one neuron to the other [20]. ACh in the synapse is broken down into choline and acetate, by acetylcholinesterase (AChE), a powerful hydrolytic enzyme [21]. Choline is taken up into cholinergic neurons by a sodium-coupled high-affinity choline transporter and is recycled into acetyl CoA [21]. Importantly, cholinergic neurotransmission is regulated by four main factors: synthesis, transport, and breakdown of ACh, and the expression level of postsynaptic cholinergic receptors [22]. Disruptions in any of these four levels of regulation could lead to reduced cognitive function. A significant piece of evidence for this is from one of the first studies on cholinergic neurotransmission, which observed that an mAChR antagonist, scopolamine, induced amnesia in humans, evidenced by impaired memory storage and retrieval as well as impaired non-memory cognitive functions [23].

### Cholinergic disruptions in AD

To understand AD beyond the neuropathological evidence of extracellular Aβ deposits and formation of NFTs intracellularly, there has been significant interest in the biochemical basis of cholinergic system disruptions in AD. These have led to important discoveries, including: (**i**) evidence for significant decline in activity of ChAT in the neocortex of AD patients [24], (**ii**) reduced uptake/transport of choline in hippocampus and cortex [25], (**iii**) reduction in release of ACh into the synapse [2], and (**iv**) profound and selective degeneration (more than 75%) of cholinergic projections originating from basal forebrain cholinergic neurons, particularly, the Ch4 neurons of the nucleus basalis of Meynert [26]. Other studies have reported loss of cholinergic neurons of other basal forebrain groups, including the Ch1 medial septal and Ch2 vertical limb of the diagonal band nuclei [27, 28]. These changes, together with validation of the role of ACh and cholinergic receptors in learning, memory, and attention [23], have led to the ‘*cholinergic hypothesis of AD*’. The hypothesis suggests that the loss of cholinergic neurotransmission in the cerebellar cortex and other regions of the brain, resulting from selective degeneration of cholinergic-expressing neurons primarily in the basal forebrain, contributes to the decline in cognitive function in AD [23,29]. Thus, drugs that can enhance cholinergic neurotransmission could be important in rescuing the learning and memory deficits in AD patients. This has been the basis for strategies to develop drugs for symptomatic treatment and disease modification in AD, with most studies showing rescue of cognitive deficits and disease modification in preclinical animal models [30–35]. In human studies, the use of drugs that cause whole-body up-regulation of cholinergic neurotransmission, or drugs that selectively target subgroups of cholinergic receptors have shown progress over the last few decades [36–40].

## Muscarinic acetylcholine receptors

Of the two groups of cholinergic receptors, the muscarinic acetylcholine receptor (mAChR) is the predominant group for ACh binding and signalling, and has been the focus of preclinical and clinical studies to develop novel therapeutic strategies for neurodegenerative and neuropsychiatric disorders [41,42]. The mAChRs are seven transmembrane (TM) class A rhodopsin-like G protein-coupled receptors consisting of five subtypes, M1–M5, which are encoded by distinct *CHRM* genes (*CHRM1–CHRM5*). Over the last decade, the crystal structures of all five mAChR subtypes have been resolved [43–46], which have confirmed previous phylogenetic analyses of a highly structurally conserved hydrophilic cavity deep within the TM unit 2–7 regions of all five mAChR subtypes [47]. ACh binds to the amino acid residues located on the outer region of this orthosteric-binding pocket through an ionic interaction between the polar, positively charged headgroup of ACh and the negatively charged aspartate residue within the TM3 [48]. Importantly, the highly conserved nature of these orthosteric pockets has been a significant limitation to designing subtype-selective ligands for these receptors.

Based on the signal transduction properties and preferential binding to different classes of G proteins, mAChRs can be grouped into two categories: M1, M3 and M5 couple to the G_q/11_ family of G proteins, leading to activation of phospholipase C and intracellular calcium (Ca^2+^) mobilisation [47], whereas M2 and M4 preferentially signal through the G_0/i_, leading to inhibition of adenylate cyclase and subsequent reduction in intracellular cytosolic cyclic adenosine monophosphate (cAMP) accumulation [48].

**Figure 1:**
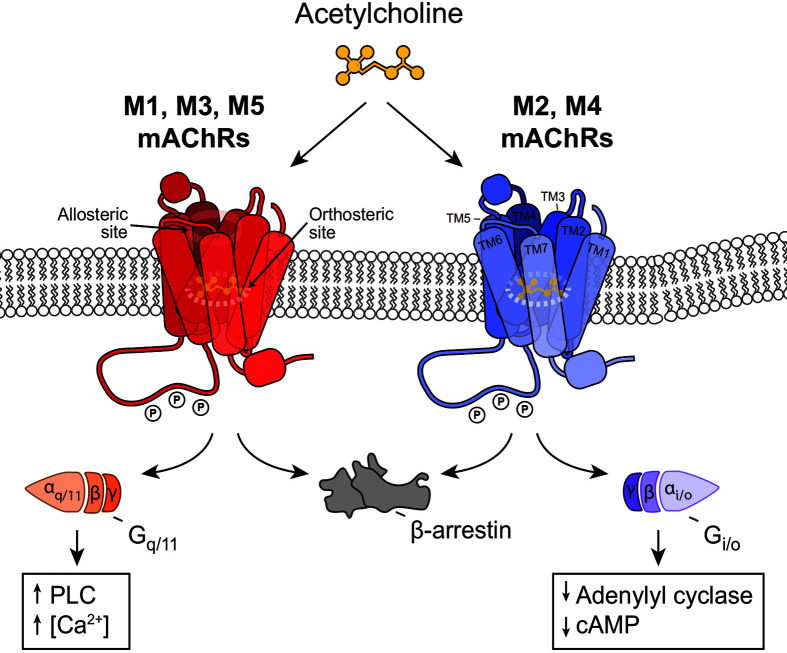
Common signalling transduction pathways of the five subtypes of the mAChR Activation of M1-, M3- or M5-mAChR results in coupling of the receptor to the G_q/11_ family of G proteins. This coupling leads to activation of phospholipase C (PLC) enzyme, with subsequent mobilisation and release of calcium (Ca^2+^) from the intracellular stores to the cytosol. Activation of M2- or M4-mAChR results in coupling to the G_0/I_ family of G proteins. This coupling leads to inhibition of adenylate cyclase enzyme, which results in decreased levels of cytosolic cAMP.

Figure 1 illustrates the common signal transduction pathways of the five mAChRs.

### Distribution and functions of mAChRs

mAChRs are widely distributed throughout the human body, and based on their location and receptor subtype, they mediate a plethora of physiological functions in the cardiovascular, renal, gastrointestinal, pulmonary, central, and peripheral nervous systems [49–55].

#### mAChRs outside of the central nervous system

An understanding of the expression and function of mAChR subtypes outside of the CNS, especially in the cardiovascular and gastrointestinal systems, is important to appreciate the adverse peripheral effects associated with targeting the receptor in AD.

In the cardiovascular system, the M2-mAChR is the predominant subtype, with significant expressions in the atria and ventricular myocardium regions of the human heart, where activation results in modulation of heart rate [56]. In the vasculature, M2-mAChR is expressed in the coronary and endothelium and primarily modulation vasodilation [57]. Moreover, other mAChR subtypes, including M5, M1, and M3 have been shown to be expressed in the vasculature and play roles in vasodilation and vasoconstriction when activated, suggesting a potential role in blood pressure regulation [58,59]. In rat ventricular myocytes, the M1 subtype of muscarinic acetylcholine receptor (M1-mAChR) was shown to be expressed, and when activated, it modulates intracellular calcium concentrations [60]. In addition, the M1- and M4-mAChR have been shown to be involved in regulation of potassium currents in human and rodent atrial cardiomyocytes, which could be important in atrial pathologies, including atrial fibrillation [61,62].

All the five subtypes of the mAChRs have been shown to be expressed in different tissues and cells of the GIT, including in the smooth muscle and mucosa of the intestine, colon, and stomach [52]. However, the predominant mAChRs in the GIT are the M2- and M3, which have been shown to modulate smooth muscle excitation and contraction, with consequent physiological effects including GIT motility [63].

#### Distribution and function of mAChRs in the central nervous system

The M1 subtype (M1-mAChR) makes up to 60% of the total mAChR expression in the CNS and is abundantly expressed in major forebrain areas including hippocampus, neostriatum, and cerebral cortex [64–66]. At the cellular level, M1-mAChR is abundantly expressed predominantly in postsynaptic glutamatergic and striatonigral pyramidal neurons [67,68]. The pattern of distribution and localisation of the M1-mAChR in the brain underpins its roles, including control of glutamatergic neurotransmission, synaptic plasticity, learning and memory [68,69]. The important role of M1-mAChR in cognitive function is supported by observations that genetic ablation or pharmacological inhibition of M1-mAChR signalling in rodents results in significant cognitive deficits. On the other hand, activation of the M1-mAChR rescues learning and memory deficits in preclinical models of neurodegeneration, and in human patients with CNS disorders such as schizophrenia [30–35,41,42].

The M2-mAChR subtype is widely expressed throughout the basal forebrain, thalamus, neocortex and hippocampus, and these expressions are abundant in non-cholinergic terminals in the cortex and hippocampus [64,68]. The potential of M2-mAChR antagonism to rescue cognitive deficits in neurodegeneration has been observed in rat models of cognitive impairment [70], suggesting that a combination of M2-mAChR antagonism and M1-mAChR agonism could be a better therapeutic approach to enhance learning and memory in AD. On the other hand, the observation that M2-mAChR activation inhibits dopamine release at dopaminergic terminals [67,71] suggests that M2-mAChR activation could be a potential therapeutic approach in schizophrenia, provided that the negative physiological effects of down-regulation of dopamine release are mitigated. It must be emphasised however, that M2 mAChR-knockout mice exhibit deficits in learning and recognition memory and hippocampal plasticity [72,73], indicating that antagonism, rather than complete ablation of the receptor function may be beneficial to cognition.

Among the mAChR subtypes, the M3-mAChR has the lowest level of expression in the CNS, predominantly in the hypothalamus [64,68]. Relative to M1-mAChR, the role of M3-mAChR in the CNS is largely unclear. However, a previous study has observed that M3-mAChR knockout and M3-mAChR phospho-deficient knockin (where all serine residues in the third intracellular loop of the receptor are mutated to alanine) mice exhibit learning and memory deficits [74], pointing to a potential role of M3-mAChR phosphorylation in cognitive function.

The M4-mAChR subtype is widely expressed in the corpus striatum, caudate and putamen, and is co-expressed on the striatal projection neurons with dopamine receptors [64,66], where they regulate dopamine release and inhibit dopamine D1 receptor function [75,76]. The M4-mAChR has emerged as a promising target for the treatment of cognitive and behavioural deficits in neurodegenerative and neuropsychiatric disorders. For instance, the M1/M4-preferring agonists, xanomeline has shown in human phase II trials to improve behavioural symptoms in patients with schizophrenia [41]. In addition, M4-mAChR is a potential target for treatment of Parkinson’s disease, considering that the disease is associated with significant loss of dopamine neurons projecting to the striatum, which results in an imbalance between the dopaminergic and cholinergic systems [77]. Based on this understanding, there have been development of selective M4-mAChR antagonists for the symptomatic treatment of Parkinson’s disease [78–80].

The M5-mAChR subtype is predominantly expressed in the ventral tegmental area, and by dopamine-containing neurons of the substantia nigra pars compacta. These structures provide significant dopamine innervations to the striatum and the nucleus accumbens [54,81]. The areas of the brain principally innervated by the M5-mAChR play important roles in the rewarding effects of several drugs of abuse [82], suggesting that this subtype could be a potential target for the management and treatment of drug addiction and misuse.

The M1-mAChR is the most widely studied subtype in terms of validating mAChRs as therapeutic targets for symptomatic treatment and disease modification in AD.

## M1-mAChR in AD

The M1-mAChR remains the most important mAChR subtype to target for the development of therapies for AD, due to several reasons.

First, the expression level of postsynaptic M1-mAChR remains unchanged in different regions of the AD brain when compared with normal aged brain [21,83–85]. This is an important factor in selecting a drug target. This observation however, is contrasted by other studies which have reported reduced M1-mAChR protein expression in AD brains, as well as significant reduction in coupling of the M1-mAChR to G proteins [86–88]. Nonetheless, the M1-mAChR remains a promising target for AD drug development. Second, the M1-mAChR plays important roles in processes involved in the two main hallmarks of AD pathology: formation of Aβ plaques and NFTs. There is evidence from *in vitro* studies that primary neurons from M1-mAChR knockout mice exhibit significantly increased amyloidogenic amyloid precursor protein (APP) processing [89,90], and this observation is partly due to the inhibitory role of M1-mAChR on the activity of β-secretase 1 (BACE1), as well as the enhancement of production of soluble amyloid precursor protein (sAPPα), a soluble form of APP which is neuroprotective [67]. On the other hand, activation of the M1-mAChR by agonists results in reduced tau phosphorylation in both primary cells and mouse brains [34,91]. Third, in addition to learning and memory processes, activation of M1-mAChR has been shown to improve cerebral blood flow in patients with AD [92], which is important for therapeutics development considering that disrupted blood flow is one of the most consistent and important pathological characteristics of AD pathology [93].

These observations underscore the importance of the M1-mAChR in the normal functioning of the CNS, and its role in AD, indicating the significant potential of directly activating this receptor subtype for symptomatic treatment and disease modification, without the significant adverse effects observed with whole-body up-regulation of cholinergic neurotransmission with AChEIs.

## Strategies to target M1-mAChR in AD drug development

Over the years, two main pharmacological strategies have been developed in the drug discovery process of M1-mAChR in AD. These two strategies will be discussed in detail.

### M1-mAChR orthosteric agonists for AD

Considering the critical roles that the M1-mAChR play in cognitive function, it is important to develop ligands that interact with the binding sites bound by endogenous ligand, ACh, to activate the receptor and rescue the cognitive deficits observed with disrupted cholinergic neurotransmission in AD. These ligands, known as orthosteric agonists, represent a promising therapeutic strategy. M1-mAChR orthosteric agonists have one important advantage over the use of AChEIs. In AD, there is degeneration of presynaptic neurons, which causes depletion of ACh in the synapse, such that AChEIs may not be effective in enhancing cholinergic neurotransmission [2]. The action of M1-mAChR orthosteric agonists on the other hand, are not affected by degeneration of presynaptic cholinergic neurons. Orthosteric ligands are either full or partial agonists at the M1 mAChR. A partial agonist, relative to a full agonist, have low intrinsic activity and trigger a lower response even at maximum receptor occupancy. Suggested benefits of selecting partial agonists lies in their potential to reduce the processes of agonist-induced desensitisation and enhanced formation of a stable binding mode [42]. These features could lead to reduced adverse cholinergic effects at clinically relevant concentrations.

Over the last few decades, there has been significant progress in the development of orthosteric agonists for AD, and several of these ligands are in various stages, ranging from testing in preclinical models to advanced human trials. Some of the M1-mAChR orthosteric agonists that have shown procognitive effects in preclinical models include AF267B, WAY-132983, CDD-0102A, and SPP1 [94–97].

Xanomeline, developed by Elli Lilly, preferentially binds to the M1- and M4-mAChR subtypes. This ligand confers improvement in cognitive function and rescue of behavioural deficits in both rodent and human studies [40,98–100]. These results underpin studies that have assessed the efficacy of xanomeline in rescuing behavioural deficits in schizophrenia [101]. Consequently, phase II human trials for xanomeline in AD patients failed to progress due to associated cholinergic adverse effects, including nausea, diarrhoea, hypersalivation, and vomiting [40]. These adverse effects have been attributed to the relatively poor selectivity of xanomeline for M1-mAChR, with off-target binding to peripheral M2- and M3-mAChRs [102]. Of significant importance is the recent report of a phase II human trial from Karuna Therapeutics, which shows that combining xanomeline with trospium, a peripherally restricted pan-mAChR antagonist, resulted in significant improvements in behavioural deficits in patients with schizophrenia, assessed on the Positive and Negative Syndrome Scale (PANSS) [41]. A previous phase I study involving healthy volunteers had reported that incidence of cholinergic adverse effects were halved in the xanomeline-trospium (KarXT) group, compared with xanomeline only group [103]. These reports raise the potential of trialling a combination of M1-mAChR orthosteric agonist and peripherally restricted antagonists of other mAChR subtypes in mild to moderate AD patients.

Furthermore, Sosei Heptares, together with their collaborating academic scientists used a Structure-Based Drug Design (SBDD) approach to develop an M1-mAChR orthosteric partial agonist, HTL9936, which shows significant procognitive effects in preclinical animal models, including mice, rats, dogs, and non-human primates [42]. In elderly human subjects, HTL9936 shows a low profile of cholinergic adverse effects and activates the centres of learning and memory [42].

These recent developments have restored confidence in this area of AD drug research, and the potential of M1-mAChR orthosteric agonists in AD.

Table 1 summarises the status of the key M1-mAChR orthosteric agonists at various stages of human clinical trials for symptomatic treatment of cognitive deficits in AD.

**Table 1:** Summary of M1-mAChR orthosteric agonists in clinical development for AD

Compound	Source	Clinical stage	Comments	References
Xanomeline	Elli Lilly	Phase II (discontinued)	- M1/M4-preferring full agonist - Adverse cholinergic effects reported	[40,98–100]
HTL9936	Sosei Heptares	Phase I (safety, tolerability, pharmacokinetic and pharmacodynamic study; completed)	- M1-selective partial agonist	[42]
HTL0018318	Sosei Heptares	Phase Ib (safety and tolerability; completed)	- M1-selective partial agonist - Phase IIb trials for dementia with Lewy bodies	[104,105]
Talsaclidine	Boehringer Ingelheim	Phase II (efficacy and safety study; completed)	- M1-selective full agonist - Talsaclidine showed no improvement in cognition in patients with mild to moderate AD	[106]
AF102B	SnowBrand Pharmaceuticals	Phase I (discontinued)	- Reduces Aβ levels in AD patients, first M1 agonist to have such effect in humans - No evidence of improvement in cognition	[94,107]

### M1-mAChR allosteric ligands for AD

The highly conserved orthosteric-binding sites of the five mAChR subtypes continues to be a major challenge to the development of M1-mAChR-selective orthosteric agonists. An alternative approach is to target the allosteric binding sites of the M1-mAChR. Structural studies have shown that the M1-mAChR and other mAChR subtypes have allosteric-binding sites that are normally located above the orthosteric-binding site, in an extracellular vestibule [43,108]. Importantly, there is significant diversity in the amino acid residues that are important for binding of allosteric ligands into the allosteric pocket of the M1-mAChR [109–111], and this provides an important strategy for designing and developing allosteric ligands with greater receptor subtype-selectivity.

#### Pharmacology of M1-mAChR allosteric ligands

The binding of a ligand to the allosteric pocket of the M1-mAChR leads to significant changes in receptor conformation, and this allosteric ligand-bound receptor can be viewed as being a *‘novel’receptor type* with a unique behaviour [112]. Depending on the type of allosteric ligand, binding to the M1-mAChR can result in several changes. First, this binding can have a direct effect on the affinity of the orthosteric-bound ligand. This is thought to be induced by the localised alterations in the site topography of the orthosteric-binding site, due to the conformational change triggered by the binding to the allosteric site, and this change either enhances or reduces the binding affinity of the orthosteric ligand [113,114]. Second, a broader change in conformation of the M1-mAChR by an allosteric ligand can result in a change in the overall downstream signalling efficacy of the orthosteric-bound ligand [115,116], either enhancing or reducing the potency of the orthosteric ligand. Third, some allosteric ligands can exert direct allosteric agonism, independent of the presence or activity of the orthosteric ligand [113,114].

**Figure 2:**
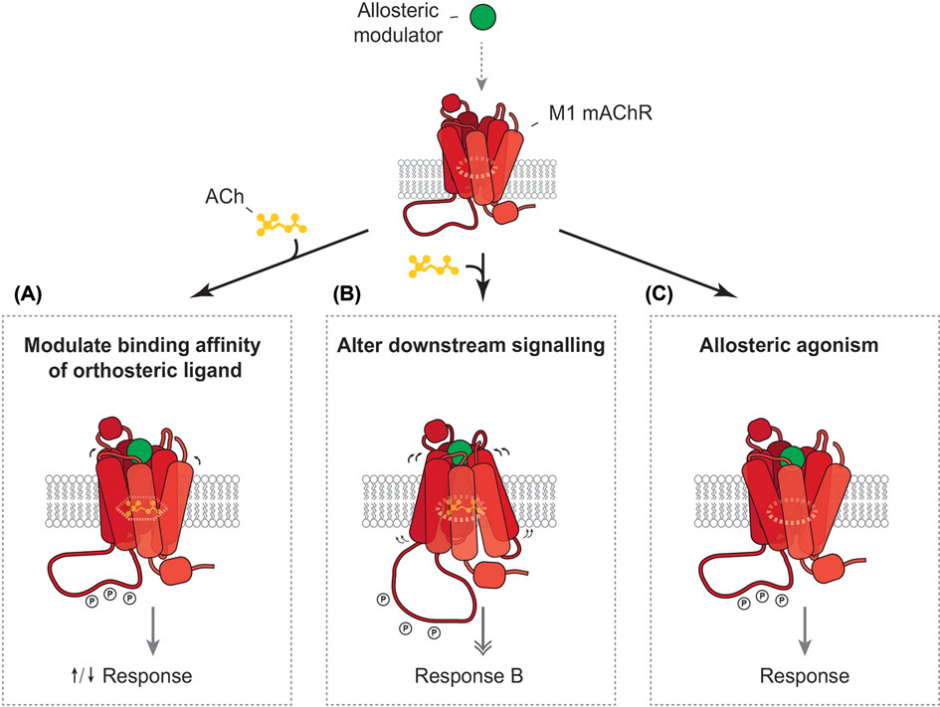
Allosteric modulation at the M1-mAChR Binding of a ligand to the allosteric pocket of the M1-mAChR can have three effects. (**A**) A conformational change in the receptor, leading to a change in the binding affinity of the orthosteric-bound ligand, in this case, ACh for the receptor. (**B**) A broader conformational change, leading to a change (increase or decrease) in the potency of ACh toward a particular signalling response. (**C**) A direct agonist effect exerted by the allosteric ligand independent of the presence of the orthosteric-bound ligand, ACh.

Figure 2 illustrates the modes by which allosteric ligands exert their effect at the M1-mAChR.

#### Types of allosteric ligands at the M1-mAChR

Based on the type of allosteric modulation exerted, allosteric ligands can be classified into four groups.

- *Allosteric agonists* bind and interact with the M1-mAChR at a site that is distinct from the orthosteric-binding site of the endogenous ligand, and these agonists can activate the receptor independent of the presence of the orthosteric-bound ligand [67,117]. Allosteric agonists present with two promising characteristics; first, there is potential for subtype-selectivity, with less possibility of off-target adverse cholinergic effects. Second, the process of activating the receptor independent of the presence of the endogenous ligand (ACh) shows promise for use in up-regulating cholinergic neurotransmission in AD, at stages where ACh is depleted.
- *Neutral allosteric ligands* (NALs) bind to the allosteric site of the M1-mAChR without exerting any effect on the downstream signalling or causing a change in the activity of the bound orthosteric ligand. An example of an M1-mAChR NAL is Compound 13b, which does not affect the potency of ACh to induce accumulation of IP1 [116].
- *Positive allosteric modulators* (PAMs) bind to the allosteric pocket to enhance the affinity and/or efficacy of the orthosteric-bound ligand. Based on their intrinsic agonist activity, PAMs can be categorised into two groups.

‘Pure’ PAMs enhance the pharmacological properties of the orthosteric-bound ligand, but they exert no direct effect on the receptor. *‘Pure’* PAMs are only active in the presence of an orthosteric ligand [108,113]. However, the notion of ‘pure’ PAMs is disputed based on evidence that the type of assay, strength of the signal arising from coupling to the pathways being measured, and the system in which the signal is being measured could all affect the measure of intrinsic agonist activity [113,118]. Thus, to take full advantage of these ‘pure’ PAMs in AD drug discovery, there is the need for better systems to measure the intrinsic activity of PAMs.

On the other hand, PAM-agonists, in addition to enhancing the pharmacological activity of the bound orthosteric ligand, exert their own direct intrinsic agonist effects on the receptor, independent of the bound orthosteric ligand [116,119].

- *Negative allosteric modulators* (NAMs) bind to the allosteric site of the receptor to reduce the pharmacological binding and/or potency of the bound orthosteric ligand. As with PAMs, NAMs can be ‘pure’ NAMs or NAM-agonists [112].

#### Advantages of targeting M1-mAChR allosteric sites in AD drug development

Theoretically, allosteric ligands have several advantages over orthosteric ligands. First, because the allosteric-binding sites of the five mAChR subtypes have less conserved amino acid sequences compared to the orthosteric sites, there is potential for development of subtype-selective M1-mAChR ligands, which could reduce off-target cholinergic adverse effects. It must be emphasised, however, that there has been reported evidence of on-target adverse effects with some M1-mAChR allosteric ligands, particularly the PAM-agonists [32]. Secondly, with ‘pure’ PAMs, there is the advantage of avoiding these on-target adverse effects. Binding of orthosteric ligands to the M1-mAChR results in either a complete activation or inhibition of the receptor and its downstream signalling and consequent physiological response [114]. Depending on the concentration of the ligand and the receptor occupancy, there is a risk of on-target desensitisation, overdose or tolerance, and side effects. On the other hand, there is a ‘ceiling effect’ in downstream signalling and physiological effects induced by allosteric ligands, particularly because of the degree of modulation (positive or negative) by allosteric modulator [120]. This degree of modulation is dependent on several factors, including the degree of cooperativity, such that once all the orthosteric-binding sites are occupied, there is a limit on the intensity of modulation and action [113]. Third, there is potential for allosteric modulators, particularly PAM-agonists to potentiate the activity of the depleted levels of ACh in latter stages of the AD process, while binding to and activating the postsynaptic M1-mAChRs which have been shown to remain relatively intact in AD. Another important advantage of targeting allosteric sites of the M1-mAChR lies in the conservation of the spatiotemporal pattern of signalling [121]. Because ‘pure’ PAMs require the presence of an orthosteric ligand to exert their effects on the receptor, this could be exploited in controlling the activity of PAMs to areas where there is ACh, in a manner of spatial and temporal specification [113,122].

## M1-mAChR positive allosteric modulators for AD

With significant deficits in cholinergic neurotransmission in AD, M1-mAChR positive allosteric modulators (M1-PAMs) remain promising options for rescuing learning and memory deficits. Several pharmaceutical and academic establishments have developed M1-PAMs, with some of these ligands reaching different stages of drug development.

### M1-PAMs from Merck

#### BQCA

One of the first reported exemplar M1-PAMs was 1-(4-methoxybenzyl)-4-oxo-1,4-dihydroquinoline-3-carboxylic acid, benzyl quinolone carboxylic acid (BQCA). This was found to be highly selective for the M1-mAChR [123,124]. Moreover, BQCA reversed scopolamine-induced memory deficits and amphetamine-induced hyperlocomotion in rodents [124,125]. Interestingly, in addition to these effects, there is evidence of disease-modifying properties of BQCA, characterised by significant increase in survival of terminally diseased prion neurodegeneration mice [30]. Recently, a global proteomics analysis of the hippocampus of prion neurodegenerative mice have shown that chronic dosing with BQCA reduces the levels of proteins shown to be involved in neuroinflammation, neurodegeneration and aberrant complement activation, thereby unravelling the biochemical changes that underpin the disease-modifying actions of BQCA [31]. Despite the positive observations in preclinical studies, BQCA failed to progress into clinical trials due to its poor solubility, high plasma protein-binding properties, and poor brain penetrance [124]. Merck has produced other M1-PAMs which are structurally similar or based on the scaffold of BQCA.

#### PQCA

1-((4-cyano-4-(pyridine-2-yl)piperidin-1-yl)methyl-4-oxo-4H-quinolizine-3-carboxylic acid (PQCA), like BQCA, has been shown to rescue memory deficits in rodent models of scopolamine-induced memory deficits as well as reverse deficits in recognition memory in aged Tg2576 APP mice, a mouse model of AD [126,127]. In both studies, there was no evidence of adverse cholinergic effects, further supporting the potential of these M1-PAMs for symptomatic treatment of memory deficits in AD without accompanying adverse effects.

#### MK-7622

This is the most-advanced M1-PAM from Merck and has shown reversal of scopolamine-induced memory deficits in non-human primates and in humans [128]. In a phase II proof-of-concept clinical trial in patients with mild to moderate AD, 45 mg of MK-7622 was used as an adjunctive therapy, but this dose did not improve cognition in these patients [129]. Interestingly, 16% of these patients experienced adverse cholinergic effects, particularly diarrhoea, compared with only 6% in the control group. These adverse effects could be attributed to the robust intrinsic agonist activity of MK-7622 observed in *in vitro* assays, and the observation that MK-7622 induces behavioural convulsions and seizures in wildtype, but not M1-knockout mice [33,130]. These suggest that M1-PAMs with robust agonist activity may not be viable precognitive compounds and could induce on-target cholinergic adverse effects.

**Figure 3:**
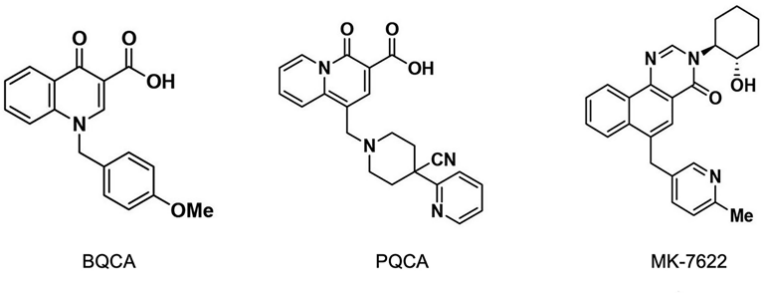
Chemical structures of M1-mAChR allosteric ligands developed by Merck PQCA and MK-7622 are based on the chemical scaffold of BQCA.

Figure 3 shows the structures of the common M1-PAMs from Merck.

### M1-PAMs from Pfizer

#### PF-06767832

Considered a PAM-agonist in most systems, this ligand is highly selective for the M1-mAChR in functional assays in recombinant systems. It has good cooperativity, and displays good brain penetrance [131]. In studies in rats, PF-06767832 reversed scopolamine-induced deficits in Morris water maze, and attenuated amphetamine-induced deficits in pre-pulse inhibition. This ligand, however, induced convulsions, and gastrointestinal and cardiovascular adverse effects in these rats. It was shown that these effects were due to on-target M1-activation, rather than off-target activation of M2- or M3-mAChR [131].

#### PF-06827443

This is a potent, highly selective M1-PAM with procognitive effects and good brain penetrance [132,133]. However, in both rats and dogs, the ligand-induced cholinergic adverse effects and convulsions at therapeutic indices similar to PF-06767832. Studies using native brain tissue and moderate-to-high expression-transfected cell systems showed that, PF-06827443 displayed significantly robust agonist activity, likely explaining the accompanying cholinergic affects [132].

#### PF-06764427

Classified as a PAM-agonist, this ligand displayed robust agonist activity in cells lines and mouse prefrontal cortex, which may underlie the convulsions it induced in mice [130].

**Figure 4:**
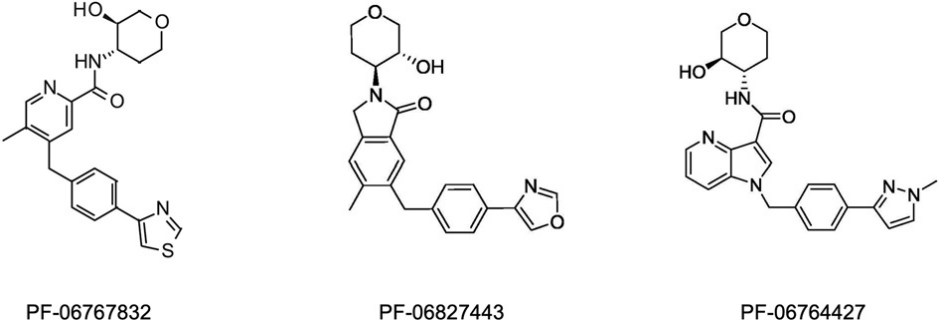
Chemical structures of M1-mAChR allosteric ligands developed by Pfizer PF06827443 and PF-06764427 are based on the chemical scaffold of PF-06767832.

Figure 4 shows the structures of the common M1-PAMs from Pfizer.

### M1-PAMs from Vanderbilt University

The Conn and Lindsley research groups at Vanderbilt University in Nashville, Tennessee, have been important in the development of small molecule M1-PAMs.

#### VU139

This is the most advanced M1-PAM from Vanderbilt, with reports from the university and data from conference proceedings showing that the ligand is in a phase I human trial with the end aim of slowing memory loss in patients with AD and schizophrenia (see: https://www.vumc.org/ccm/sites/default/files/VU319%20ARTICLE%20FROM%20VU.pdf).

#### VU0486846

A highly selective M1-PAM shown to exhibit good brain penetrance with little to no intrinsic agonist activity. Studies show reversal of risperidone-induced memory deficits in rats without any adverse cholinergic effects [32]. In terminal murine prion neurodegenerative model, acute administration of VU0486846 rescued learning and memory deficits, while chronic administration resulted in significantly reduced levels of misfolded prion protein and extended the lifespan of these mice. Proteomics analysis showed corrections in expression of markers of neuroinflammation, aberrant mitochondrial and complement function [31]. In addition, in an APP mice model of AD, chronic VU0486846 administration resulted in reduced levels of Aβ, rescue of cognitive deficits, and a shift in APP processing from amyloidogenic to non-amyloidogenic cleavage [134].

#### VU0453595

This M1-PAM has been shown to lack significant agonist activity in a number of assays, including in calcium mobilisation assay in cells expressing rat M1-mAChR [132]. This lack of intrinsic agonist activity may underlie the little to no adverse effects observed in rodents dosed with the ligand [132]. In a previous study, VU0453595 enhanced the long-term depression (LTD) mediated by the mAChR agonist, carbachol in prefrontal cortex slices from mice [135]. Moreover, in mice, acute administration of VU0453595 rescued phencyclidine-induced impairment in cognition and social interactions [135], thereby suggesting a potential role of this M1-PAM in both cognitive and behavioural deficits associated with neurodegenerative and neuropsychological disorders.

#### VU6004256

This ligand has been profiled as a PAM-agonist, with the agonist activity relatively weaker compared with other PAM-agonists such as PF-06764427. In mice, intraperitoneal administration of VU6004256 resulted in enhanced recognition memory in the novel object recognition test [130], suggesting a potential to assess of this ligand in neurodegenerative models for rescue of cognitive function. Moreover, unlike other PAM-agonists, administration of higher dose (100 mg/kg) did not result in observable behavioural seizure activity [130], which could be due in part to the relatively low agonist activity.

**Figure 5:**
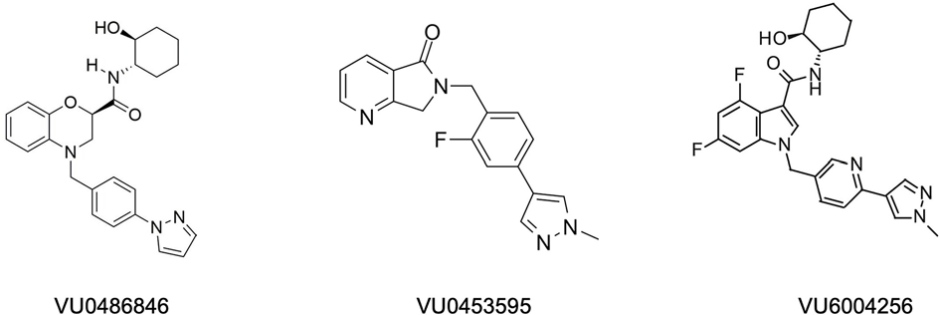
Chemical structures of M1-mAChR allosteric ligands developed by Vanderbilt University VU0453595 and VU6004256 are based on the chemical scaffold of VU0486846.

Figure 5 shows the structures of the common M1-PAMs from Vanderbilt.

Other groups that have M1-PAMs in different stages of development include Takeda (TAK-071) and Monash Institute of Pharmaceutical Sciences (MIPS) with their MIPS class of M1-PAMs [116,136].

Table 2 summarises some of the important M1-mAChR allosteric modulators and their properties.

**Table 2:** Summary of M1-mAChR allosteric modulators and their relevance to AD

Compound	Source	Ligand properties	Potential role in AD	Comments	Reference(s)
BQCA	Merck	PAM-agonist	- Rescues cognitive deficits in rodents - Increases survival and reverses neuroinflammation in terminal neurodegenerative mice	- Poor solubility - High plasma protein-binding properties	[30,31,123,124]
PQCA	Merck	PAM-agonist	- Rescues scopolamine-induced cognitive deficits in rodents - Reverses deficits in recognition memory in AD mice model - Wider margin between doses leading to cognitive effects and adverse effects	- Exhibits favourable CNS penetration in rodents	[126,127]
MK-7622	Merck	PAM-agonist	- Rescues cognitive deficits in rodents and non-human primates	- Phase II trials discontinued - No observed improvement in cognition in patients with mild to moderate AD - Induces M1-dependent adverse effects	[33,127–130]
PF-06767832	Pfizer	PAM-agonist	- Rescues scopolamine-induced cognitive deficits in rodents	- Induces on-target adverse effects	[131,133]
PF-06827443	Pfizer	PAM-agonist	- Good brain penetrance - Induces procognitive effects in rodents	- Induces adverse cholinergic effects	[130,133]
PF-06764427	Pfizer	PAM-agonist	- No observed procognitive effects in mice - Reduces hyperlocomotion in mice and rats	-Induces convulsions in rodents	[130]
VU139	Vanderbilt University	Not available	- Not available	- Phase I clinical trials underway	-
VU0486846	Vanderbilt University	PAM	- Rescues cognitive deficits in AD mice and prion terminal neurodegeneration mice - Reduces levels of misfolded prion and amyloid-beta proteins in AD mice and prion terminal neurodegeneration mice - Increases survival and reverses neuroinflammation in terminal neurodegenerative mice		[31,32,133]
TAK-071	Takeda	PAM	- Rescues scopolamine-induced cognitive deficits in rodents - Wider margin between doses leading to cognitive effects and adverse effects	- Phase I clinical trials (concluded)	[33,136]

## Leveraging M1-mAChR signalling bias to achieve beneficial clinical outcomes in AD

Depending on the ligand, binding to the M1-mAChR results in stabilisation of different conformation states, which can induce differential/preferential activation of the receptor signalling pathways, or collateral efficacy. This phenomenon, known as signalling bias, may define the physiological outcomes of the receptor activation [137,138]. To determine if a ligand is biased towards a particular signalling pathway, the activity of the ligand (affinity, efficacy, potency) is calculated for a range of different pathways, which are then compared to calculations for these same pathways for a reference ligand (e.g., ACh for M1-mAChR) which has been shown in the past to activate all these known coupled pathways [137,138]. Signalling bias represents an attractive strategy for the development of M1-mAChR ligands that may be biased towards pathways which results in reduced adverse effects, while giving all the important therapeutic outcomes.

The physiological relevance of ligand-induced signalling bias is illustrated in an *in vivo* phosphoproteomics study of the κ opioid receptor (KOR), which unravelled the signalling pathways that are associated with desired (antipruritic and anticonvulsant properties) and undesired (aversion, dysphoria, psychotomimetic) outcomes of activating the KOR [139]. The study provided data to link the gap between observations from *in vitro* pharmacology and *in vivo* works by showing that KOR agonists that are known to elicit differential signalling profiles induced differential dynamic phosphorylation of synaptic proteins.

There is evidence for the potential benefits of M1-mAChR signalling bias in AD drug discovery. Bradley and colleagues [140] used a M1-mAChR phosphorylation-deficient (PD) mouse to show that the M1-mAChR partial agonist, pilocarpine, is biased towards G protein-dependent signalling over phosphorylation-dependent signalling *in vivo*, based on the observation that pilocarpine induces approximately six-fold cortical increase in phosphoinositide turnover over basal, compared with approximately two-fold increase induced by GSK1034702, a bitopic M1-mAChR agonist. Thus, M1-mAChR ligands that are biased towards phosphorylation or arrestin-dependent signalling could have efficacy in exerting clinically beneficial outcomes, while minimising adverse effects.

Interestingly, two M1-PAMs; PF-06767832 and PF-06827443, which have been shown to induce seizures in mice causes significant increase in striatal inositol monophosphate levels *in vivo* [131,133]. Inositol monophosphate is downstream of the G protein-dependent signalling, and this suggests that these two M1-PAMs may have biased G protein-dependent signalling over phosphorylation-dependent signalling, resulting in these seizures.

**Figure 6:**
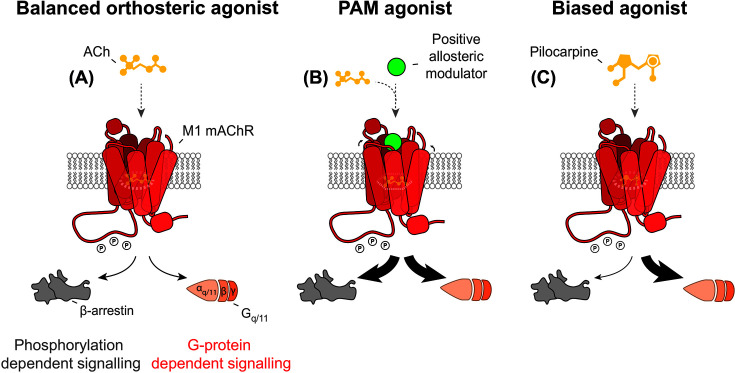
Ligand-induced signalling bias at the M1-mAChR (**A**) ACh binds to the orthosteric pocket of the M1-mAChR to activate the receptor, resulting in induction of conformational changes within the receptor. This leads to coupling of the receptor to G proteins and β-arrestin with equivalent potencies, and subsequent activation of multiple signalling pathways downstream of G proteins and β-arrestin. (**B**) Binding of an ‘unbiased’ PAM-agonist to the M1-mAChR allosteric pocket potentiates coupling to G proteins and β-arrestin with equivalent potencies, but results in enhanced activation of multiple signalling pathways downstream of G proteins and β-arrestin. (**C**) Pilocarpine is biased towards G protein signalling over β-arrestin signalling, hence binding to the orthosteric pocket of the M1-mAChR results in receptor conformations that promote coupling to G proteins with greater potency than to β-arrestin. This results in activation of signalling pathways downstream of G proteins over β-arrestin.

Figure 6 illustrates the phenomenon of M1-mAChR signalling bias.

## What is the ideal M1-mAChR drug for AD?

The pathological processes involved in AD begin several years before the clinical signs of the disease appear. This stage is known as the preclinical AD stage. The three stages after diagnosis, namely early stage (mild), middle stage (moderate), and later stage (severe) can occur within a timeframe of approximately 20 years depending on several factors. Considering that defective cholinergic signalling, evidenced by degeneration of cholinergic neurons, depleted ACh levels, and reduced activity of ChAT correlates with progression of AD and accompanying symptoms [141,142], strategies for developing the ‘ideal’ M1-mAChR drug should be approached with this in mind.

In most cases, in the preclinical stage, most patients exhibit MCI, with evidence of neuropathological, biochemical, and molecular features of AD [143]. At this stage, M1-PAMs that display little to no agonist activity (‘pure’ PAMs) could be the ideal option to potentiate the affinity and potency of existing ACh, without the risk of adverse effects. Pure M1-PAMs could also be ideal for the early (mild) stages of the disease. Moreover, with significant disruptions in ChAT activity and depleted ACh levels in the middle (moderate) stage of the disease, M1-mAChR PAM-agonists could be used to up-regulate signalling of the depleted ACh while simultaneously activating the receptor. Thus, it is important to understand the subtle biochemical and pharmacological profiles of M1-mAChR PAM-agonists to develop ligands that will elicit these effects with minimal to no adverse effects.

In the later (severe) stages of the disease when ACh levels are significantly depleted, M1-mAChR orthosteric agonists may be ideal as ACh replacement to directly activate the receptor to enhance signalling and neurotransmission with consequent improvement in cognitive function. Another option in the late stage of the disease is the use of bitopic M1-mAChR ligands. These are ligands that can simultaneously bind to the orthosteric- and allosteric-binding pockets of the receptor owing to their special chemical scaffold, consisting of an orthosteric and allosteric-binding moieties linked together by a linker [144]. This approach will benefit from the development of M1-mAChR-specific orthosteric and bitopic ligands with favourable signalling bias pharmacology, which will reduce the risk of cholinergic adverse effects.

It must be emphasised that for some patients with AD, some of these disease stages may overlap, and late diagnosis of the disease could also mean that the opportunity to use some of these drugs may be lost.

More importantly, there is the emergence of M1-mAChR ligands which, in addition to treating the cognitive and behavioural deficits (symptoms) of the disease, could also modify the progression of the disease by reducing the levels of disease-causing proteins [30,31,134,145]. These new class of AD ligands, when developed into drugs, will be very important for rescuing cognitive impairment and/or slowing down progression of different stages of the disease. Furthermore, in selecting or developing an M1 mAChR ligand for AD, there needs to be an important risk-benefit assessment to ensure that the best possible strategies and options are available to patients.

**Figure 7:**
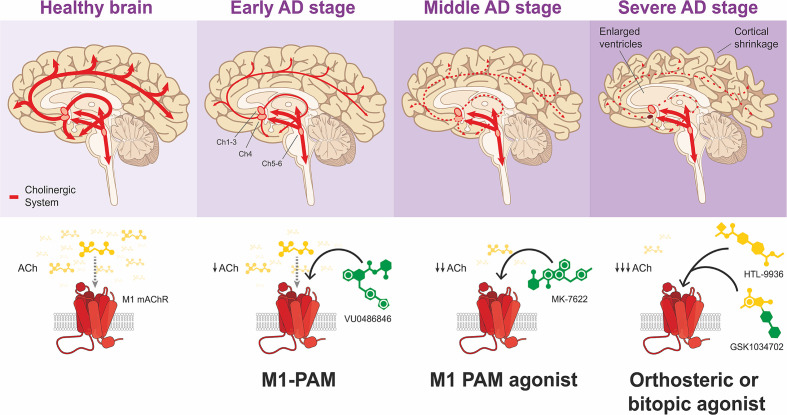
Linking M1-mAChR drug development to pathological stages of AD In the early stages of the disease when there is gradual loss of cholinergic neurons and modest depletion of ACh as well as MCI, use of ‘pure’ M1-PAMs may be a good strategy to potentiate the actions of ACh and improve cognitive function with minimal risk of adverse effects. With progression to the moderate stages of the disease, there is relatively increased loss of ACh coupled with more pronounced disruptions to cognitive and behavioural functions. At this stage, the use M1-mAChR PAM-agonists to simultaneously potentiate the actions of ACh and directly activate the receptors may be an ideal strategy. In the severe stages of AD, there is significant depletion of ACh and severe loss of cognitive function. Orthosteric or bitopic ligands could be ideal in ‘replacing’ the actions of the depleted ACh to improve cognitive function. In developing these strategies, it is important to assess the risk-benefit profile of these ligands in the context of the patient.

Figure 7 illustrates the stages of different stages of AD and the ‘ideal’ M1 mAChR ligand for treatment of these stages.

## Conclusion

The need to develop effective drugs for the treatment of AD is unmet, in the face of increasing cases of the disease worldwide. Over the last few decades, there has been different strategies to target the major pathological hallmarks of the disease. The strategy to target the disease-causing proteins in AD to slow disease progression and improve cognition has resulted in the approval of Aducanumab, an Aβ monoclonal antibody by the US FDA.

Another strategy has been to develop subtype-selective drugs to target the M1-mAChR. This has seen the development of orthosteric ligands such as xanomeline and recently, HTL9936 from preclinical models to human trials. Allosteric ligands are in the early stages of development, but with the promising data seen in preclinical studies, it is hoped that more of these ligands will progress into human trials in the near future.

Furthermore, one important consideration for future development of M1-mAChR drugs for AD is the concept of signalling bias. This will be important in the development of drugs that will confer the beneficial effects (pro-cognition and disease modification) while avoiding the adverse cholinergic effects seen with previous medications. Despite this concept being in the early stages, it represents a promising strategy.

The different stages of AD, presenting with varying degrees of neuropathology and disrupted signalling processes may influence the type of M1-mAChR drug that will be required. More importantly, a thorough assessment of the benefits and risks of using a particular drug should be considered in all stages of development of M1-mAChR drugs for AD.

In conclusion, there has been a slow, yet significant progress in the strategy to target the M1-mAChR in AD, and the next few years will be challenging, but exciting in this area of AD drug development. The hope is that some of these drugs will move from the benchtop to the bedside sooner, rather than later.

## Data Availability

This is a Review Article which contains no original data.

## Abbreviations

ACh: acetylcholine
AChE: acetylcholinesterase
AChEI: acetylcholinesterase inhibitor
AChR: acetylcholine receptor
APP: amyloid precursor protein
AD: Alzheimer’s disease
Aβ: β-amyloid peptide
BQCA: 1-(4-methoxybenzyl)-4-oxo-1,4-dihydroquinoline-3-carboxylic acid, benzyl quinolone carboxylic acid
cAMP: cyclic adenosine monophosphate
ChAT: choline acetyltransferase
CNS: central nervous system
GIT: gastrointestinal tract
KOR: κ opioid receptor
mAChR: muscarinic acetylcholine receptor
MCI: mild cognitive impairment
M1-mAChR: M1 subtype of muscarinic acetylcholine receptor
M1-PAM: M1-mAChR positive allosteric modulator
NAL: neutral allosteric ligand
NAM: negative allosteric modulator
NFT: neurofibrillary tangle
NMDA: N-methyl-D-aspartate
PAM: positive allosteric modulator
PQCA: 1-((4-cyano-4-(pyridine-2-yl)piperidin-1-yl)methyl-4-oxo-4H-quinolizine-3-carboxylic acid
TM: transmembrane
